# Effort-reward imbalance and overcommitment in employees in a Norwegian municipality: a cross sectional study

**DOI:** 10.1186/1745-6673-3-9

**Published:** 2008-04-30

**Authors:** Bjørn Lau

**Affiliations:** 1 National Institute of Occupational Health, Oslo, Norway

## Abstract

**Background:**

The aim of this study was to validate a Norwegian version of the Effort-Reward Imbalance Questionnaire (ERI-Q).

**Methods:**

One thousand eight-hundred and three employees in a medium-sized Norwegian municipality replied to the ERI-Q, and health-related variables such as self-reported general health, psychological distress, musculoskeletal complaints, and work-related burnout were examined.

**Results:**

Sound psychometric properties were found for this Norwegian version of the ERI-Q. When the two dimensions of ERI and overcommitment were analyzed in four types of employees, the results showed that employees characterized by a combination of high values on ERI and overcommitment had more unfavorable health scores than others. Employees with low effort-reward and overcommitment scores had more favorable health scores. Employees with scores on the overcommitment and the effort-reward scales that are supposed to have opposite effects on health (that is, the combination of low overcommitment with a high effort-reward score and *vice versa*), had health scores somewhere in between the two other groups.

**Conclusion:**

Satisfactory psychometric properties were found for most of the latent factors in the ERI-Q. The findings also indicate that it may be fruitful to explore health conditions among employees with different combinations of effort-reward and overcommitment.

## Background

According to the effort-reward imbalance (ERI) model by Siegrist et al. [1], effort at work is part of a social contract that is reciprocated by adequate reward. Rewards are distributed by three transmitter systems: esteem, career opportunities, and job security. Failed reciprocity between efforts and rewards may enhance the activation of the autonomic nervous system and influence the risk of coronary heart disease [2-4]. According to the model, adverse health effects can also be triggered by an individual's exhaustive coping style, known as overcommitment. More specifically, this model consists of three hypotheses [5]: (1) *The ERI hypothesis*: The mismatch between high effort and low reward (no reciprocity) produces adverse health effects, (2) *The overcommitment hypothesis*: A high level of personal commitment (overcommitment) increases the risk of reduced health (even when the ERI is absent), and (3) *The interaction hypothesis*: Relatively higher risks of reduced health are expected in people who are characterized by conditions (1) and (2).

High effort and low reward conditions have repeatedly been shown to be positively associated with the incidence of coronary events [6-10]. Overcommitment has also been shown to be associated with increased risks of cardiovascular disease (CVD) (for a review, see van Vegchel et al. [11]). Corresponding support has not been found for the interaction hypothesis regarding CVD risk factors or CVD symptoms [11].

The ERI model and its hypotheses have also been investigated in terms of self-reported health and well-being (for a review, see Tsutsumi and Kawakami [12] and van Vegchel et al. [11]). The ERI has been found to be related to self-reported health [13-16], poor well-being [17], and depression [18,19]. Overcommitment has been found to be associated with musculoskeletal pain [20], depression [21], psychosomatic complaints [13], and self-reported health in men [14]. Support for the interaction hypothesis is inconsistent. For instance, a higher risk for emotional burnout due to ERI in overcommitted employees was found in one study [17], but not in another [21].

In a comparison of results from five European studies (Belgium, France, Germany, Sweden, and the UK), variations of the components in the ERI model were reviewed according to types of occupation, education, age, and gender [1]. In three countries, the effort scale measurements were higher in men than in women, whereas a reverse tendency was found in the UK study. Lower effort was associated with increased age in two studies with a high proportion of elderly subjects. Mean effort was significantly higher among better-educated groups in four samples, and a similar nonsignificant tendency was observed in a smaller German sample. Reward did not differ according to gender in a consistent way, but there was a tendency of higher sores among older employees and especially in men. A positive association of reward with degree of education was observed in two samples. A clear-cut gradient was observed with higher reward scores among higher employment grades. Men and women aged 45–54 generally had the highest overcommitment scores, and employees with higher education tended to exhibit higher overcommitment scores.

This study has three aims. Because the effort-reward model has not been systematically examined in Norway, the standardized self-administrated questionnaire for measuring ERI (ERI-Q) [1] was translated into Norwegian and answered by employees in a Norwegian municipality. The first aim of this study was to examine the factor structure of this instrument with confirmatory factor analyses. Secondly, the differences in mean values of the components in the ERI instrument, according to gender, age, education, and occupation, were examined. Thirdly, to explore criterion validity, the ERI hypothesis, the overcommitment hypothesis and the interaction hypothesis were tested in relation to self-reported health, psychological distress, musculoskeletal complaints, and work-related burnout.

Siegrist does not specify whether the interaction hypothesis refers to additive main effects or to a synergistic effect. A synergetic understanding of an interaction effect is that the level of a moderator variable influences the relationship between the independent variables and the dependent variable. In line with such a view, we would expect the associations between effort-reward imbalance and the health variables included in this study to be strongest among employees with high scores on overcommitment. Most studies have tested for the interaction hypothesis on a variable level using regression analysis. However, because we were also interested in employees with scores on the overcommitment and effort-reward scales that are supposed to have opposing effects on health (that is, the combination of low overcommitment with high effort-reward score and *vice versa*), we also divided the respondents into four groups according to combinations of high and low scores on the overcommitment scale and the effort-reward scale, respectively. This resulted in four groups of employees: *Relaxed employees, Struggling employees, Exaggerated employees*, and *Despaired employees*.

*Relaxed employees *are nonovercommitted employees that receive sufficient reward when effort is taken into consideration. *Struggling employees *are employees that are not overcommitted, but experience an imbalance in effort compared with reward. *Exaggerated employees *are overcommitted employees working in an environment where effort is reciprocated with reward. *Despaired employees *are overcommitted employees subjected to a working environment where their effort in work is not matched by the reward they receive. We would expect to find despaired employees to have more unfavorable scores on the health-related variables compared with others. Further, we expected to find favorable health scores among *relaxed employees*. We were also interested in the groups whose scores on the overcommitment and the effort-reward scales are supposed to have opposing effects on health (that is, struggling employees and exaggerated employees).

## Methods

All employees in a middle-sized municipality in Norway were invited to participate in a study of their psychosocial workplace environment. The research design was based on a web-based questionnaire. Researchers at the National Institute of Occupational Health received a list of all the employees in this municipality. In order to generate identification numbers and *"Subject Access Codes" *for the web-based questionnaire, the list contained names, gender, age, social security number, department worked in, and the International Standard Classification of Occupations (ISCO-88). All employees were sent a personal written invitation to participate in the research project through the internal mail at their workplace. The invitation consisted of general information regarding the purpose of the study, and their personal access code to the web-based questionnaire. The data were collected within a three-week period in the spring of 2007.

### Participants

Of the 2712 employees working in the municipality, 1803 participated, giving a response rate of 66.5%. As can be seen in Table 1, the response rate among younger employees was lower than in other age groups, the response rate among employees working in the department of administration was higher than in other departments, and response rate differed across different occupational groups.

**Table 1:** Description of the sample

		Invited	Participated frequency	Participated percent	χ^2^
Total		2712	1803	66.5	
Gender	Men	560	368	65.7	0.2
	Women	2150	1433	66.7	
Age	-29	152	87	57.2	18.1**
	30–39	570	350	61.4	
	40–49	781	540	69.1	
	50–59	826	573	69.4	
	60-	381	251	65.9	
Department	Health and Social	1184	773	65.3	24.7***
	Culture and Leisure	106	68	64.2	
	Administration	78	69	88.5	
	School and Kindergarten	1031	688	66.7	
	Technical	301	193	64.1	
Occupation groups	Low-skilled blue-collar workers	135	68	50.4	48.7***
	High-skilled blue-collar workers	77	43	55.8	
	Low-skilled white-collar workers	1093	678	62.0	
	High-skilled white-collar workers	1405	1012	72.0	

Note: * *p *< 0.05, ** *p *< 0.01, *** *p *< 0.001

### Measurements

#### Occupation groups

Based on the ISCO-88 codes, four categories of employees were distinguished: 1) low-skilled blue-collar workers (ISCO codes 8 and 9), mainly helpers and cleaners in offices and other establishments; 2) high-skilled blue-collar workers (ISCO codes 6 and 7), including road workers, construction workers, and landscape gardeners; 3) low-skilled white-collar workers (ISCO codes 4 and 5), including nursing and care assistants, child-care workers, home helpers, and secretaries; and 4) high-skilled white-collar workers (ISCO codes 1, 2, and 3), including primary education teaching-associated professionals, nurses, social workers, and production and operations department managers in education, health, and social security. Participation levels, according to these categories, are shown in Table 1.

#### Effort-reward imbalance model

**Figure 1:**
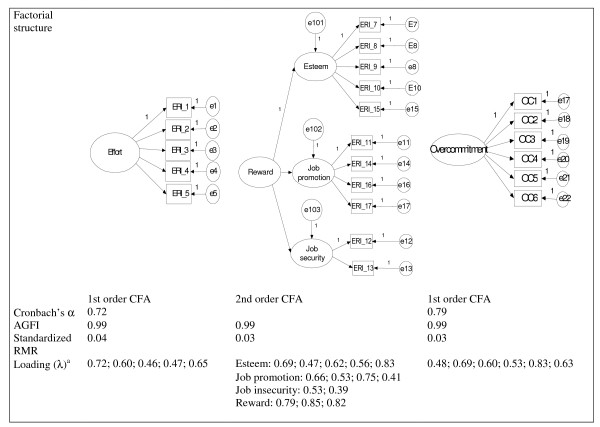
Factorial structure and goodness of fit measures of the three components of the ERI model.

The standardized self-administrated questionnaire for measuring the ERI (ERI-Q) [1] was translated from English into Norwegian by a back-translation process. Five items measured effort, while reward was measured with three components: esteem (five questions), job promotion (four questions), and job security (two questions). All items are shown in Figure 1, and Table 2. Items on the effort scale were answered in two steps. First, subjects agreed or disagreed on whether the item content described a typical experience of their work situation. Those who agreed that it was typical were asked to evaluate the extent to which these conditions produce strain, using a four-point rating scale. The final options were: 1 = "does not apply"; 2 = "does apply, but not strained"; 3 = "does apply and somewhat strained"; 4 = "does apply and strained"; and 5 = "does apply and very strained". The 11 items measuring reward were framed similarly, although the coding was reversed, so that the lower the summary scores for reward, the higher the subjective ratings of distress due to low reward.

**Table 2:** All items of the different scales in the Effort-Reward Questionnaire (ERI-Q).

"Effort" scale	"Reward" scale	"Overcommitment" scale
Time pressure (ERI_1)	Component esteem	Overwhelmed by pressure (OC1)
Interruptions (ERI_2)	Respect from superiors (ERI_7)	Think about work (OC2)
Responsibility (ERI_3)	Respect from colleagues (ERI_8)	Relax and "switch off" work (OC3)
Pressure to work overtime (ERI_4)	Adequate support (ERI_9)	Sacrifice too much for job (OC4)
Increasing demands (ERI_5)	Unfair treatment (ERI_10)	Work still on mind (OC5)
	Respect and prestige at work (ERI_15)	Trouble sleeping at night (OC6)
	Component job promotion	
	Job promotion prospects (ERI_11)	
	Adequate position (ERI_14)	
	Adequate work prospects (ERI_16)	
	Adequate salary/income (ERI_17)	
	Component job security	
	Undesirable change (ERI_12)	
	Job security (ERI_13)	

The ratio of effort (numerator) and reward (denominator) quantifies the amount of ERI, as the ERI increases with increasing values of the ratio. The effort-reward ratio was calculated as follows: effort/reward × correction factor (factor correcting for the difference in the numbers of items of the two scales). More details of the psychometric properties of these scales are provided in the Results section.

Overcommitment at work (OC) was measured with the short form of the Intrinsic Effort Scale [1]. Five items focus on the "inability to withdraw from work" and one item focuses on "disproportionate irritability." On a four-point rating scale (1 = strongly disagree, 2 = disagree, 3 = agree, 4 = strongly agree) the participants answered the related questions, which are reported in Figure 1. More details of this scale's psychometric properties are provided in the Results section.

#### Self-reported poor health

In order to measure overall individual health, the question "How is your health in general?" was asked, with the following response categories: 1 = excellent, 2 = very good, 3 = good, 4 = rather good, and 5 = poor. This categorical variable has been shown to be a very good predictor variable of other outcomes, such as subsequent use of medical care or of mortality (see, e.g., Idler and Benyamini [22]). In logistic regression analyses, a dichotomized version of this question was used. The response categories four and five were combined to indicate poor self-rated health, giving a total of 10.9% with self-reported poor health.

#### Musculoskeletal complaints

Musculoskeletal complaints were measured with the "musculoskeletal pain" scale from the Subjective Health Complaint Inventory [23]. This scale measures the extent to which respondents had been affected by pain in the neck, upper back, lower back, arms, feet, shoulders, or migraine during the last month. Response categories were: 0 = not at all, 1 = a little, 2 = some, and 3 = serious. A principal component analysis with a varimax rotation confirmed a one-factor solution of the scale. Cronbach's alpha was found to be 0.81. In logistic regression analyses, a dichotomized version of scale was used. Values above one (24.1% of the respondents) were taken to indicate musculoskeletal complaints.

#### Psychological distress

Psychological distress (anxiety and depression symptoms) during the previous 14 days was assessed with the SCL-5, a shortened version of the Hopkins Symptom Checklist-25 [24]. The SCL-5 consists of five questions (feeling fearful, feeling hopeless about the future, nervousness or shakiness inside, feeling blue, worrying too much about things), each with four answer options: 1 = not at all, 2 = a little, 3 = quite a bit, and 4 = extremely. The index was scored as the mean of the item scores. The SCL-5 index has, in different studies, been shown to correlate strongly (*r *> .90) with the SCL-25 index [24,25], which is a valid measure of psychological distress [26,27]. In our data, a principal component analysis with a varimax rotation confirmed a one-factor solution of the scale. Cronbach's alpha was 0.84. In logistic regression analyses, a dichotomized version of this scale was used. The cut-off point was set at the value of two [24,25], giving a total of 6.8% of cases.

#### Work-related burnout

Burnout was measured with the work-related burnout scale from the Copenhagen Burnout Inventory [28]. This scale consists of seven items on exhaustion, attributed to work in general. The questions are: Is your work emotionally exhausting?, Do you feel burnt out because of your work?, Does your work frustrate you?, Do you feel worn out at the end of the working day?, Are you exhausted in the morning at the thought of another day at work?, Do you feel that every working hour is tiring for you?, and Do you have enough energy for family and friends during leisure time? Response categories for the first three questions were: to a very high degree, to a high degree, somewhat, to a low degree, and to a very low degree. Response categories for the last four questions were: always, often, sometimes, seldom, and never/almost never. The score for the last question was reversed. Scoring was conducted according to the procedure outlined in Kristensen et al. [28]: To a very high degree or always = 100, to a high degree or often = 75, somewhat or sometimes = 50, to a low degree or seldom = 25, and to a very low degree or never/almost never = 0. The total score on the scale is the average of the scores on the items. A principal component analysis with a varimax rotation confirmed a one-factor solution of the scale. Cronbach's alpha was 0.85. In logistic regression analyses, a dichotomized version of this scale was used. The cut-off point was set at the value of 50, giving a total of 13.6% of cases.

### Statistics

Psychometric properties of the ERI-Q (internal consistency, factorial structure) were tested by calculating Cronbach's alpha and with confirmatory factor analysis, respectively. Confirmatory factor analyses were estimated by the unweighted least squares method, which does not presume multivariate normal distribution.

To test for differences in the mean values of the components in the ERI instrument, according to gender, age, education, and occupation, a series of General Linear Models univariate analyses of variance were computed. Gender, age, education, and occupation were entered simultaneously as independent variables in separate analyses for the different components of the ERI model. Pairwise comparisons were tested simultaneously with post-hoc Sidak tests.

Multivariate logistic regression analyses were performed to test *the ERI hypothesis *(that a mismatch between high effort and low reward is associated with adverse health), and *the overcommitment hypothesis *(that a high level of personal commitment is associated with reduced health). Effort-reward scores higher than one and the upper tertile scores of overcommitment were defined as high-risk scores. In these analyses, gender, age, education, and occupation were controlled for.

Because logistic regression analysis reduces data information quite substantially, linear regression analyses were also conducted in order to test the ERI hypothesis, the overcommitment hypothesis and the interaction hypothesis. In order to test the ERI hypothesis and the overcommitment hypothesis, effort-reward ratio and overcommitment were entered simultaneously in a series of regression analyses. The interaction hypothesis was tested by entering multiplicative terms (effort-reward ratio*overcommitment) to the models evaluated in the first step. All independent variables were centered in these analyses, and they were controlled for gender, age, education, and occupation.

To test the hypothesis that relatively higher risks of reduced health are expected in people who are characterized by experiencing failed reciprocity between efforts and rewards and overcommitment, participants were assigned to four groups according to their scores on the overcommitment scale and their effort-reward ratio score. These groups were named *relaxed employees *(low on overcommitment and low on ERI), *struggling employees *(low on overcommitment and high on effort-reward), *exaggerated employees *(high on overcommitment and low on effort-reward), and *despaired employees *(high on both overcommitment and effort-reward). Scores above the 90^th ^percentile on the overcommitment scale were characterized as high, and scores higher than one on the effort-reward ratio scale were typified as high. The GLM univariate procedure in SPSS was used to test for mean differences in the health-related variables between these four groups. In these analyses, gender, age, education, and occupation were controlled for. Pairwise comparisons were tested simultaneously with post-hoc Sidak tests, which adjusts the significance level for multiple comparisons.

Amos 7 was used to compute the confirmatory factor analyses, while all other analyses were conducted with SPSS version 15.

### Ethics and approvals

The data for research purposes was anonymous as all names and personal ID numbers were omitted. The study was conducted in accordance with the World Medical Association Declaration of Helsinki and with permission from the Data Inspectorate of Norway.

## Results

Figure 1 describes the psychometric properties of the instrument used to measure the effort-reward and overcommitment factors. All Cronbach's alpha values were satisfactory (alpha > 0.70) with α 0.72 on the "effort" scale, α 0.78 on the "reward" scale, and α 0.76 on the "overcommitment" scale. According to this, item responses obtained for each scale highly correlate with each other, indicating high internal consistency.

With respect to the confirmatory factor analyses, the Adjusted Goodness of Fit Index (AGFI) and the Standardized Root Mean Square Residual (SRMR) were within respective limits on all three scales (AGFI > 0.90 and SRMR < 0.05). Finally, all items measuring the respective constructs of effort, reward, and overcommitment, with the exception of the item "My job security is poor", loaded on the scales to a sufficiently high degree (i.e., > 0.40), thus supporting the notion of unidimensionality of these scales. Overall, this information indicates a good model fit according to established standards.

As shown in Table 3, the prevalence of persons having higher scores on the efforts scale compared with the reward scale (when adjusted for differences in the number of items on the scales), that is, the ERI ratio, did not significantly differ according to gender, age, education, or occupation groups. However, some differences were found on the different components of the ERI model. The youngest employees were more likely to have low effort values compared with employees in their fifties. High-skilled white-collar workers reported higher scores on the effort dimension than all other occupation groups. High-skilled white-collar workers had higher levels of overcommitment than low-skilled blue-collar workers and low-skilled white-collar workers.

**Table 3:** Descriptive statistics of the components in the Effort-reward model. Estimated mean and standard error (SE) of Effort, Reward, Overcommitment, and ERI ratio according to gender, age, education, and occupation group.

		Effort		Reward		Over-commitment		ERI			
		Estimated mean	SE	Estimated mean	SE	Estimated mean	SE	Estimated mean	SE	Percent with high score (> 1)	χ^2^
Total		11.7	4.2	47.8	6.5	12.1	3.4	0.6	0.3	5.4 (n = 96)	
Gender	Men	11.1	0.3	47.5	0.5	11.6	0.2	0.5	0.0	6.6 (n = 24)	1.2
	Women	10.8	0.3	48.3	0.4	11.8	0.2	0.5	0.0	5.2 (n = 72)	
Age	-29	10.2a	0.5	47.9	0.8	11.9	0.4	0.5	0.0	3.6 (n = 3)	3.7
	30–39	10.8	0.3	48.0	0.5	11.8	0.3	0.5	0.0	4.1 (n = 14)	
	40–49	11.3	0.3	47.7	0.4	11.5	0.2	0.5	0.0	5.3 (n = 28)	
	50–59	11.6a	0.3	47.3	0.4	11.9	0.2	0.6	0.0	6.8 (n = 38)	
	60-	10.9	0.3	48.5	0.5	11.4	0.3	0.5	0.0	5.3 (n = 13)	
Education	Comprehensive school	10.7	0.4	48.7	0.6	11.5	0.3	0.5	0.0	3.8 (n = 5)	1.0
	Secondary/vocational school	10.5	0.3	47.4	0.4	11.6	0.2	0.5	0.0	5.8 (n = 39)	
	College degree	11.0	0.3	47.6	0.5	11.9	0.3	0.5	0.0	5.6 (n = 45)	
	Higher university degree	11.7	0.4	47.8	0.7	11.9	0.4	0.6	0.0	4.6 (n = 7)	
Occupation groups	Low-skilled blue-collar	10.7a	0.6	48.0	0.9	11.1a	0.5	0.5	0.0	4.8 (n = 3)	2.6
	High-skilled blue-collar	9.9b	0.7	47.9	1.0	11.6	0.5	0.5	0.0	0 (n = 0)	
	Low-skilled white-collar	10.9c	0.3	47.3	0.4	11.6b	0.2	0.5	0.0	5.6 (n = 37)	
	High-skilled white-collar	12.3abc	0.2	48.4	0.4	12.5ab	0.2	0.6	0.0	5.6 (n = 56)	

Note: Values with same letter are significantly different at the 0.05 level.

As can be seen in Table 4, the effort-reward ratio was associated with all the health-related variables in logistic regression analyses. The strongest associations were found with work-related burnout (OR: 7.1; 95% CI: 4.4–11.3) and psychological distress (OR: 4.3; 95% CI: 2.5–7.5). Weaker associations were found with musculoskeletal complaints (OR: 2.9; 95% CI: 1.8–4.7) and self-rated poor health (OR: 1.8; 95% CI: 1.0–3.1).

**Table 4:** Effort-reward ratio, overcommitment and self reported health. Effort-reward ratio and overcommitment entered simultaneously in adjusted logistic regression analyses (adjusted for gender, age, education, and occupation) to predict self-rated poor health, musculoskeletal complaints, psychological distress, and work-related burnout

	Self-rated poor health		Musculoskeletal complaints		Psychological distress		Work-related burnout	
	OR	95% CI	OR	95% CI	OR	95% CI	OR	95% CI
Effort-reward ratio								
**≤ 1**	**1**		**1**		**1**		**1**	
> 1	1.8	1.0–3.1	2.9	1.8–4.7	4.3	2.5–7.5	7.1	4.4–11.3
**Overcommitment**								
Low*	**1**		**1**		**1**		**1**	
High*	2.3	1.5–3.6	1.8	1.3–2.7	4.7	3.0–7.3	5.4	3.7–7.8

* Cut-off point set at the 90^th ^percentile.

Overcommitment was also associated with all the health-related variables. Again, the strongest associations were found with work-related burnout (OR: 5.4; 95% CI: 3.7–7.8) and psychological distress (OR: 4.7; 95% CI: 3.0–7.3), and weaker associations with musculoskeletal complaints (OR: 1.8; 95% CI: 1.3–2.7) and self-rated poor health (OR: 2.3; 95% CI: 1.5–3.6).

**Figure 2:**
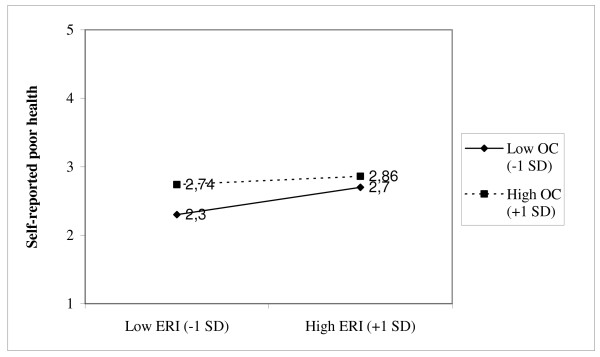
Simple slopes of the interaction for self-reported poor health on effort-reward ratio at different levels of overcommitment (one *SD *below the mean and one *SD *above the mean).

**Figure 3:**
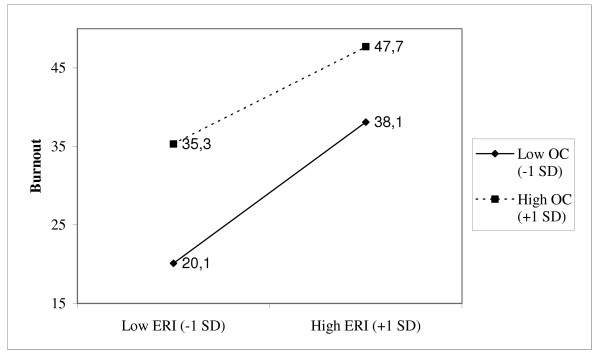
Simple slopes of the interaction for work related burnout on effort-reward ratio at different levels of overcommitment (one *SD *below the mean and one *SD *above the mean).

As shown in Table 5, the ERI hypothesis and the overcommitment hypotheses were also supported in the linear regression analyses. An interaction effect between effort-reward and overcommitment was only marginally supported with one percent additional variance explained of self-reported poor health and work-related burnout, respectively. That the interaction terms were significant in these analyses means that the slopes of the regression lines of the health variables on effort-reward ratio depend on the level of overcommitment. Simple slopes of self-reported poor health and work related burnout, respectively on effort-reward ratio at different levels of overcommitment (1 SD below the mean, and 1 SD above the mean) are shown in Figure 2 and Figure 3. The regression lines in these figures indicate that the dependence of these health scores on effort-reward ratio changed as a function of the level of overcommitment. However, the interactions were not in the predicted direction. As indicated in both Figure 2 and Figure 3, an increase in effort-reward ratio was associated with a smaller increase in both predicted self-reported poor health and work related burnout among employees with high scores on overcommitment compared to employees with low scores on overcommitment. However, as also indicated in these figures, the highest disadvantageous health scores were found among overcommited employees with high scores on effort-reward ratio.

**Table 5:** Linear regression models predicting self-reported health measures by effort-reward ratio, overcommitment and the interaction between effort-reward ratio and overcommitment (adjusted for gender, age, education, and occupation)

		Self-rated poor health			Musculoskeletal complaints			Psychological distress			Work-related burnout		
Step		β	Adj R^2^	Adj R^2 ^Change	β	Adj R^2^	Adj R^2 ^Change	β	Adj R^2^	Adj R^2 ^Change	β	Adj R^2^	Adj R^2 ^Change
**1**	**Effort-reward ratio**	.14*			.25*			.19*	.		.45*		
	**Overcommitment**	.17*	.09*	.05*	.13*	.15*	.10*	.32*	.21*	.21*	.37*	.43*	.43*
**2**	Effort-reward ratio* Overcommitment	-.09*	.10*	.01*	-.04	.15*	.00	.06	.21*	.00	-.10*	.44*	.01*

Note: * *p *< 0.01

As shown in Table 6, *relaxed employees *reported better mental health, less work-related burnout, and fewer musculoskeletal complaints than all the other groups. They also reported better general health than strugglers and exaggerators. *Struggling employees *reported poorer general health, more musculoskeletal complaints, poorer mental health problems, and more work-related burnout, compared with relaxed employees. However, they had better mental health and less work-related burnout than the despaired. *Exaggerated employees *reported poorer general health, and more musculoskeletal complaints, mental health problems, and work-related burnout, than relaxed employees. On the other hand, they had fewer musculoskeletal complaints, better mental health, and less work-related burnout than the despaired. *Despaired employees *reported more mental problems and work-related burnout than all the other groups. They also had more musculoskeletal complaints than the relaxed and exaggerated, but not the strugglers. However, in spite of these complaints they did not perceive their health as worse than the other groups.

**Table 6:** Health related variables according to combinations of overcommitment and effort-reward ratio. Estimated mean and standard error (SE) of health-related variables according to combinations of Overcommitment (OC) and Effort-reward imbalance ratio (ERI ratio), controlled for gender, age, education, and occupation groups.

	Relaxed employees		Struggling employees		Exaggerated employees		Despaired employees	
	Low OC Low ERI		Low OC High ERI		High OC Low ERI		High OC High ERI	

	N = 1524		N = 55		N = 136		N = 40	
	Mean	SE	Mean	SE	Mean	SE	Mean	SE
Self-rated poor health	2.39ab	0.06	2.77a	0.14	2.76b	0.09	2.53	0.16
Musculoskeletal complaints	0.67abc	0.04	1.12a	0.09	0.95bd	0.06	1.32cd	0.10
Mental problems	1.30abc	0.03	1.75ad	0.06	1.72be	0.04	2.09cde	0.07
Work-related burnout	29.13abc	0.93	49.32ad	2.23	46.88be	1.55	60.35cde	2.56

Note: Values with same letter are significantly different at the 0.01 level.

## Discussion

This is the first study to investigate the psychometric properties of a Norwegian version of the ERI-Q. Satisfactory psychometric properties were found for most of the latent factors in this instrument when used on employees in a medium-sized Norwegian municipality. However, the item "My job security is poor" loaded weakly on the dimension "job security". This might reflect that job security is high among the respondents in this study due to permanent employment and little tradition for dismissal for economic reasons in the public sector in Norway. Consequently, a latent factor measuring *poor job security *among respondents employed in a municipality will not necessarily be connected to either a perception or expectation of experiencing undesirable change in the work situation ("I have experienced or I expect to experience an undesirable change in my work situation") or a perception of poor job security. However, such a latent factor would be more likely to consist of these two items in a private sector working population.

In this study, 5.4% of the participants had higher mean scores on the effort factor than the reward factor, indicating an ERI. Most typically, employees with lower socioeconomic positions report higher ERI at work [29]. However, this was not supported in our study. Both the ERI ratio and the continuous ERI score were alike, according to gender, age, education, and occupational groups. On the other hand, in line with other studies, we found high-skilled white-collar workers to have the highest score on the effort scale [1]. This might indicate that employees in this group are in positions where they are expected to achieve on a high level. However, high efforts in this group might also be confounded by an active life orientation [30] that predisposes them to both aspire for higher positions and to make more effort at work. The finding that this group also had the highest score on the overcommitment factor gives some support to such a notion.

In contrast to other studies [1], we found a tendency toward higher reported levels of effort with increased age; the difference between employees in their fifties and employees in their twenties was significant. This indicates that perceived strain associated with work increases with age, and may reflect that employees in their fifties are expected to perform the same amount of work as their younger colleagues.

Both the ERI hypothesis and the overcommitment hypothesis were supported in the multivariate logistic regression analyses and in the linear regression analyses in this study. However, these findings need to be replicated in prospective studies to indicate causal relationships.

With self-reported poor health and work related burnout as the outcome variables, effort-reward ratio showed a significant but weak interaction with overcommitment in separate linear regression analyses. However, these interactive effects accounted for only an additional 1% of the variance in both health scores. Furthermore, in both cases moderator analyses showed that effort-reward ratio interacted with overcommitment in the opposite direction than expected. That is, the associations between effort-reward ratio and these health variables were weaker among employees with high scores on overcommitment compared to employees with low scores on overcommitment. Consequently, the interaction hypothesis was not supported in the linear regression analyses.

However, when assigning employees to different groups according to their scores on the effort-reward and overcommitment scales, we found, as expected, despaired employees to have more unfavorable health scores than others. This finding supports the interaction hypothesis, which states that the combination of overcommitment and high effort-reward score is especially detrimental. The hypothesis that relaxed employees would have more favorable health scores was also supported.

Employees with scores on the overcommitment and the effort-reward scales that are supposed to have opposite effects on health (that is, the combination of low overcommitment with a high effort-reward score and *vice versa*), had health scores somewhere in between the two other groups. In line with the results from the logistic regression analyses and the linear regression analyses, these results showed that both high effort-reward and overcommitment are independently associated with adverse health scores.

Categorizing respondents into these groups complemented existing knowledge about the effects of these factors by giving a graphic, comprehensive, and differentiated understanding about possible health effects based on the individuals' experience of their working environment and excessive motivation to work. In addition, such a group division can be of practical importance when choosing occupational intervention to reduce health complaints based on occupational stress. Strugglers will possibly profit from most of the interventions that make the working environment less strenuous or more rewarding in terms of recognition, job security, or career opportunity. Exaggerators, on the other hand, would probably benefit more from individual counseling aimed at reducing their overcommitment. The despaired would probably benefit most from a combination of both intervention forms.

However, there are some disadvantages to splitting up these groups. Using combinations of high versus low scores on the effort-reward and overcommitment scales, respectively, may simplify interpersonal variability by reducing the individuals' positions on continuous scales to merely two possibilities each, thereby disregarding small but important differences. Converting interval scales to ordinal scales also reduces the predictive power. However, this is first and foremost a problem when using the groups, based on the effort-reward and overcommitment scores together with other variables, to predict an outcome. In such instances, the explained variance of the effort-reward and overcommitment combinations will be less than if the dimensions were used as interval scales.

### Limitations and strengths of the study

This study relied on a large survey with a reasonable response rate. Although the sample size was large, the present data were female-dominated and from the public sector. Therefore, the findings should be interpreted with caution until they are validated in studies using other samples. The study was based on a cross-sectional design; therefore, we do not claim that the observed associations are evidence of a causal relationship. Although high ERI may lead to an increased likelihood of the co-occurrence of unfavorable health, such unsatisfactory self-reported job conditions might also reflect bad health. Poor mental health or burnout symptoms might contribute to a perception of work conditions as tedious and straining. The associations between ERI, its components, and co-occurring self-reported health appeared even when confounders such as age, gender, occupational position, and education were controlled for. However, because individual factors, such as negative affectivity or personality, were not included in this study, confounding influence from these factors cannot be excluded.

## Conclusion

This is the first study to investigate the psychometric properties of a Norwegian version of the ERI-Q. Satisfactory psychometric properties were found for most of the latent factors. When assigning employees to different groups according to their scores on the effort-reward and overcommitment scales, overcommitted employees with high effort-reward scores had especially detrimental health scores, while employees with low scores on both overcommitment and effort-reward had the most favorable health scores. Employees with scores on the overcommitment and the effort-reward scales that are supposed to have opposite effects on health (that is, the combination of low overcommitment with a high effort-reward score and *vice versa*), had health scores somewhere in between the two other groups. Categorizing respondents into these groups can be of practical importance when choosing occupational intervention to reduce health complaints based on occupational stress.

## Competing interests

The author declares that they have no competing interests.

## Authors' contributions

BL was involved in conception and design, acquisition, analysis and interpretation of data and writing of the manuscript.

## References

[B1] Siegrist J, Starke D, Chandola T, Godin I, Marmot M, Niedhammer I, Peter R (2004). The measurement of effort-reward imbalance at work: European comparisons. Soc Sci Med.

[B2] Siegrist J (1996). Adverse health effects of high-effort/low-reward conditions. J Occup Health psychol.

[B3] Siegrist J (2002). Reducing social inequalities in health: work-related strategies. Scand J Public Health Suppl.

[B4] Siegrist J, Peter R (1996). Threat to occupational status control and cardiovascular risk. Isr J Med Sci.

[B5] Siegrist J, Perrewe P, Ganster D (2002). Effort-reward imbalance at work and health. Research in Occupational Stress and Well Being.

[B6] Bosma H, Peter R, Siegrist J, Marmot M (1998). Two alternative job stress models and the risk of coronary heart disease. Am J Public Health.

[B7] Niedhammer I, Goldberg M, Leclerc A, David S, Bugel I, Landre MF (1998). Psychosocial work environment and cardiovascular risk factors in an occupational cohort in France. J Epidemiol Community Health.

[B8] Peter R, Alfredsson L, Knutsson A, Siegrist J, Westerholm P (1999). Does a stressful psychosocial work environment mediate the effects of shift work on cardiovascular risk factors?. Scand J Work Environ Health.

[B9] Peter R, Siegrist J (2000). Psychosocial work environment and the risk of coronary heart disease. Int Arch Occup Environ Health.

[B10] Siegrist J, Peter R, Junge A, Cremer P, Seidel D (1990). Low status control, high effort at work and ischemic heart disease: Prospective evidence from blue-collar men. Soc Sci Med.

[B11] van Vegchel N, de Jonge J, Bosma H, Schaufeli W (2004). Reviewing the effort-reward imbalance model: Drawing up the balance of 45 empirical studies. Soc Sci Med.

[B12] Tsutsumi A, Kawakami N (2004). A review of empirical studies on the model of effort-reward imbalance at work: reducing occupational stress by implementing a new theory. Soc Sci Med.

[B13] Godin I, Kittel F (2004). Differential economic stability and psychosocial stress at work: Associations with psychosomatic complaints and absenteeism. Soc Sci Med.

[B14] Niedhammer I, Tek ML, Starke D, Siegrist J (2004). Effort-reward imbalance model and self reported health: Cross-sectional and prospective findings from the GAZEL cohort. Soc Sci Med.

[B15] Pikhart H, Bobak M, Siegrist J, Pajak A, Rywik S, Kyshegyi J, Gostautas A, Skodova Z, Marmot M (2001). Psychosocial work characteristics and self rated health in four post-communist countries. J Epidemiol Community Health.

[B16] Stansfeld SA, Bosma H, Hemingway H, Marmot MG (1998). Psychosocial work characteristics and social support as predictors of SF-36 health functioning: The Whitehall II study. Psychosom Med.

[B17] de Jonge J, Bosma H, Peter R, Siegrist J (2000). Job strain, effort-reward imbalance and employee well-being: A large-scale cross-sectional study. Soc Sci Med.

[B18] Pikhart H, Bobak M, Pajak A, Malyutina S, Kubinova R, Topor R, Sebakova H, Nikitin Y, Marmot M (2004). Psychosocial factors at work and depression in three countries of Central and Eastern Europe. Soc Sci Med.

[B19] Tsutsumi A, Kayaba K, Theorell T, Siegrist J (2001). Association between job stress and depression among Japanese employees threatened by job loss in a comparison between two complementary job-stress models. Scand J Work Environ Health.

[B20] Joksimovic L, Starke D, van der Knesebeck O, Siegrist J (2002). Perceived work stress, overcommitment, and self-reported musculoskeletal pain: A cross-sectional investigation. Int J Behav Med.

[B21] van Vegchel N, de Jonge J, Meijer T, Hamers JP (2001). Different effort constructs and effort reward imbalance: Effects on employee well-being in ancillary health care workers. J Adv Nurs.

[B22] Idler EL, Benyamini Y (1997). Self-rated health and mortality: A review of twenty-seven community studies. J Health Soc Behav.

[B23] Eriksen HR, Ihlebæk C, Ursin H (1999). A scoring system for subjective health complaints (SHC). Scand J Public Health.

[B24] Strand BH, Dalgard OS, Tambs K, Rognerud M (2003). Measuring the mental health status of the Norwegian population: A comparison of the instruments SCL-25, SCL-10, SCL-5 and MHI-5 (SF-36). Nord J Psychiatry.

[B25] Tambs K, Moum T (1993). How well can a few questionnaire items indicate anxiety and depression?. Acta Psychiatr Scand.

[B26] Glass RM, Allan AT, Uhlenhuth EH, Kimball CP, Borinstein DI (1978). Psychiatric screening in a medical clinic. Arch Gen Psychiatry.

[B27] Rickels K, Garcia C-R, Lipman RS, Derogatis LR, Fisher EL (1976). The Hopkins Symptom Checklist. Assessing emotional distress in obstetric-gynecological practice. Primary Care.

[B28] Kristensen TS, Borritz M, Villadsen E, Christensen KB (2005). The Copenhagen Burnout Inventory: A new tool for the assessment of burnout. Work Stress.

[B29] Siegrist J, Marmot M (2004). Health inequalities and the psychosocial environment–two scientific challenges. Soc Sci Med.

[B30] Kouvonen A, Kivimäki M, Virtanen M, Heponiemi T, Elovainio M, Pentti J, Linna A, Vahtera J (2006). Effort-reward imbalance at work and the co-occurrence of lifestyle risk factors: cross-sectional survey in a sample of 36,127 public sector employees. BMC Public Health.

